# Radiosynthesis and Analysis of (*S*)-4-(3-[^18^F]Fluoropropyl)-L-Glutamic Acid

**DOI:** 10.1007/s11307-022-01793-3

**Published:** 2022-12-16

**Authors:** Gavin Brown, Dmitry Soloviev, David Y. Lewis

**Affiliations:** 1 Cancer Research UK Beatson Institute, Garscube Estate, Switchback Road, Glasgow, G61 1BD UK; 2 School of Cancer Sciences, University of Glasgow, Glasgow, G611QH UK

**Keywords:** [ ^18^F]FSPG, x_c_^−^ transporter, Radiochemistry, Positron emission tomography, Cancer imaging, Quality control analysis

## Abstract

**Purpose:**

(*S*)-4-(3-[^18^F]Fluoropropyl)-L-glutamic acid ([^18^F]FSPG) is an L-glutamate derivative used as a PET biomarker to assess intracellular redox status in vivo through targeting of the cystine/glutamate antiporter protein, x_c_^−^ transporter. In this report, we describe a radiosynthesis of [^18^F]FSPG for use in PET studies that address specific challenges in relation to the radiotracer purity, molar activity, and quality control testing methods.

**Procedures:**

The radiosynthesis of [^18^F]FSPG was performed using a customised RNPlus Research automated radiosynthesis system (Synthra GmbH, Hamburg, Germany). [^18^F]FSPG was labelled in the 3-fluoropropylmoiety at the 4-position of the glutamic acid backbone with fluorine-18 via substitution of nucleophilic [^18^F]fluoride with a protected naphthylsulfonyloxy-propyl-L-glutamate derivative. Radiochemical purity of the final product was determined by radio HPLC using a new method of direct analysis using a Hypercarb C_18_ column.

**Results:**

The average radioactivity yield of [^18^F]FSPG was 4.2 GBq (range, 3.4–4.8 GBq) at the end of synthesis, starting from 16 GBq of [^18^F]fluoride at the end of bombardment (*n* = 10) in a synthesis time of 50 min. The average molar activity and radioactivity volumetric concentration at the end of synthesis were 66 GBq µmol^−1^ (range, 48–73 GBq µmol^−1^) and 343–400 MBq mL^−1^, respectively.

**Conclusion:**

Stability tests using a 4.6 GBq dose with a radioactivity volumetric concentration of 369 MBq mL^−1^ at the end of synthesis showed no observable radiolysis 3 h after production. The formulated product is of high radiochemical purity (> 95%) and higher molar activity compared to previous methods and is safe to inject into mice up to 3 h after production.

## Introduction

Within oncological PET imaging, ^18^F-labelled amino acids (AA) are important radiotracers for the detection of tumour proliferation [1]. Perhaps, the most notable is *O*-(2-[^18^F]fluoroethyl)-L-tyrosine [2], although, more recently, ^18^F-analogues of glutamine and glutamic acid have become radiotracers of considerable interest. Examples include [^18^F]-(2S,4R)4-fluoroglutamine [3–7], [^18^F](2S,4S)-4-(3-fluoropropyl)glutamine [8], 4-[^18^F]fluoroglutamic acid [9], and *N*‐(2‐[^18^F]fluoropropionyl)‐L‐glutamic acid [10–13]. (*S*)-4-(3-[^18^F]fluoropropyl)-L-glutamic acid ([^18^F]FSPG) is an L-glutamate derivative used with PET for tumour detection and visualisation through targeting of the cystine/glutamate antiporter protein (xCTor SLC7A11), which is a subunit of the transport system x_c_^−^[14–16]. In clinical imaging trials, [^18^F]FSPG has demonstrated utility in the detection of brain, lung, liver, and breast cancers [17, 18]. System x_c_^−^ is important for maintaining intracellular redox homeostasis and shows increased activity in certain tumour cells compared to normal, healthy cells due to increased metabolic activity in tumour cells [19, 20]. [^18^F]FSPG has, therefore, gained interest as a PET biomarker to assess intracellular redox status *in vivo*. In the clinic, [^18^F]FSPG PET could prove valuable as a biomarker to assess x_c_^−^ transporter activity in patients with cancer to identify those suitable for oxidant and antioxidant therapies (or assess treatment response to oxidant and antioxidant therapies).

**Fig. 1.**
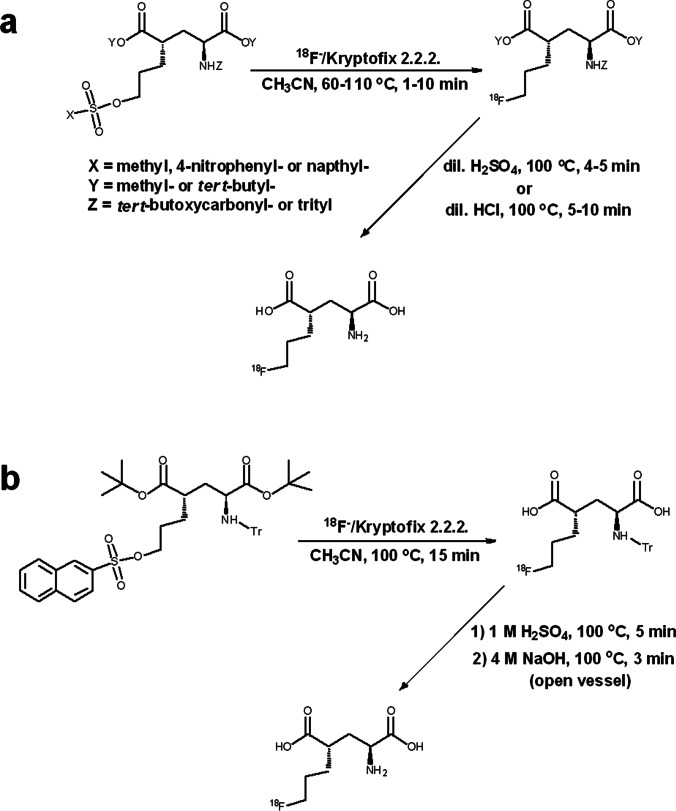
Synthesis routes to [^18^F]FSPG including **a** previously reported methods and **b** the method and conditions reported in this article.

The radiosynthesis of [^18^F]FSPG (Fig. 1) is typically carried out as a one-pot, two-step synthesis followed by purification and formulation for *i.v.* injection. In the first step, fluorine-18 incorporation into a protected precursor is achieved via nucleophilic substitution. This is followed by deprotection via acid hydrolysis to yield [^18^F]FSPG. The first reported methods (Table 1) describe the use of a precursor with a 4-nitrophenylsulphonate, leaving the group as the starting material for [^18^F]nucleophilic displacement [21, 22]. Acid hydrolysis of *O-tert*-butyl and *N-tert*-butoxycarbonyl groups then furnished [^18^F]FSPG, with radiochemical yields (RCY) from [^18^F]fluoride varying from 30 to 63% (decay corrected). A more recent method has employed the 2-naphthylsulphonate substituent as the leaving group and *O-tert*-butyl and *N*-triphenylmethyl substituents as the protecting groups [17]. For this method, the reported yield of [^18^F]FSPG from [^18^F]fluoride was 35–49% (decay corrected). While the radiolabelling and hydrolysis steps are optimised, purification of the final product has proved challenging as several methods have been applied with varied success. One of the earliest methods used a combination of reverse-phase HPLC and solid phase extraction to isolate [^18^F]FSPG with 93% radiochemical purity (RCP) [15]. Later methods have switched to cartridge purification using a combination of reverse-phase and cation-exchange stationary phases [23, 24], sometimes as part of chemical reagent cassette-based methods [17, 25]. Radiochemical purities using this approach have been as low as 90% [17, 23], although more recently values above 95% have been achieved [25, 26] with molar activities (MA) in the range of 4–48 GBq µmol^−1^ at the end of synthesis (EOS). Here, we present a non-cassette based method for the radiosynthesis of [^18^F]FSPG followed by HPLC purification and formulation via solid phase extraction that delivers a high purity product in high molar activity that is suitable for preclinical and clinical PET research.

**Table 1:** Reported reaction conditions and radiochemical analysis of [^18^F]FSPG

Entry	Precursor	Labelling temp (°C)	Labelling time (min)	Deprotection conditions	RCP (%)	RCY (%)	MA @ EOS (GBqµmol^−1^)	Reference
1	A	110	10	4.0 M HCl (150 °C, 5 min)	90–95	19–28	N/A	[22]
2	B	60–80	1–10	2.0 M HCl (100 °C, 5 min)	> 92	40–63	N/A	[21]
3	B	80	10	2.0 M HCl (100 °C, 5 min)	> 90	36 ± 17	48.47 ± 16.65	[16]
4	B	80	10	4.0 M HCl (18 °C, 5 min)	> 93	30–35	N/A	[15]
5	B	70	5	1.0 M HCl (120 °C, 10 min)	> 95	4.4 ± 2.4	14.0 ± 6.0	[24]
6	C	N/A	N/A	N/A	> 90	N/A	> 9.84	[17]
7	C	110	10	1.0 M H_2_SO_4_ (100 °C, 4 min)	> 96	38.4 ± 2.6	11.1 ± 7.7	[25]
8	C	80	10	1.0 M H_2_SO_4_ (130 °C, 4 min)	> 99	42 ± 7	4.2 ± 21.9	[26]
9	C	100	15	1.0 M H_2_SO_4_ (100 °C, 5 min)	95–97	39–43	44–73	Reported in this article

A = Di-methyl (2S,4S)-2-*tert*-butoxycarbonylamino-4-(3-methylsulfonyloxy-propyl)-pentanedioate

B = Di-*tert*-butyl (2S,4S)-2-*tert*-butoxycarbonylamino-4-(3-nitrophenylsulfonyloxy-propyl)-pentanedioate

C = Di-*tert*-butyl-(2S,4S)-2-(3-((naphthalen-2-ylsulfonyl)oxy)propyl)-4-(tritylamino)pentanedioate

## Materials and Methods

### Reagents and Materials

Kryptofix 222, potassium carbonate, 96% sulphuric acid, 37% hydrochloric acid, sodium hydroxide, sodium dihydrogen phosphate dihydrate, sodium chloride, potassium chloride, disodium hydrogen phosphate, potassium dihydrogen phosphate, and acetonitrile were purchased from Merck Life Science UK Ltd, di-*tert*-butyl-(2S,4S)-2-(3-((naphthalen-2-ylsulfonyl)oxy)propyl)-4-(tritylamino)pentanedioate (FSPG precursor) and (*S*)-4-(3-fluoropropyl)-L-glutamic acid (FSPG reference) were purchased from ABX-advanced biochemical compounds (Dresden, Germany); methanol, acetonitrile (HPLC grade), and ethanol were purchased from Rathburn Chemicals UK Ltd; QMA SepPakPlus Light cartridges were purchased from Waters UK Ltd; Chromafix PS-H^+^ (medium tube size) cartridges were purchased from Macherey–Nagel; *o*-phthalaldehyde (OPA) reagent solution reagent was purchased from Agilent Technologies UK Ltd; 0.9% saline was purchased from the Greater Glasgow & Clyde NHS Pharmacy Distribution Centre. Phosphate buffered saline (PBS), pH 7.4 was prepared as a 1-L stock solution by dissolving sodium chloride (8.00 g/137.0 mmol), potassium chloride (0.20 g/2.7 mmol), disodium hydrogen phosphate (1.44 g/10.0 mmol), and potassium dihydrogen phosphate (0.24 g/1.8 mmol) in 800 mL of water. The resultant solution was adjusted to pH 7.4 with 0.1 N HCl and then made up to a total volume of 1 L by adding water. No-carrier-added [^18^F]fluoride was obtained through the ^18^O(p, n)^18^F nuclear reaction by irradiation of 95–97 atom % oxygen-18-enriched water (purchased from Sercon UK Ltd) in a niobium target chamber (2.7-ml target volume) with a 16.4 MeV proton beam on the GE Healthcare PET trace cyclotron at the West of Scotland PET Centre. Typically, a 22 µA, 15-min target irradiation gave 15.4 GBq (16.0 GBq maximum) of [^18^F]fluoride for use at the start of a synthesis.

### Radio-HPLC Analytical Chromatography

Radio-HPLC analysis was carried out on a DionexUltimate 3000 HPLC system, consisting of an IC-3000 solvent delivery system, a WPS-3000SL-Analyt autosampler, a VWD3100 absorbance detector, a TCC3000SD column compartment, and a BioscanFC-1000 Flow countrate counter coupled to a BioscanFC-3500 PIN diode detector. The system was operated using Chromeleon software (version 6.8).

### Radiosynthesis of [^18^F]FSPG

**Fig. 2.**
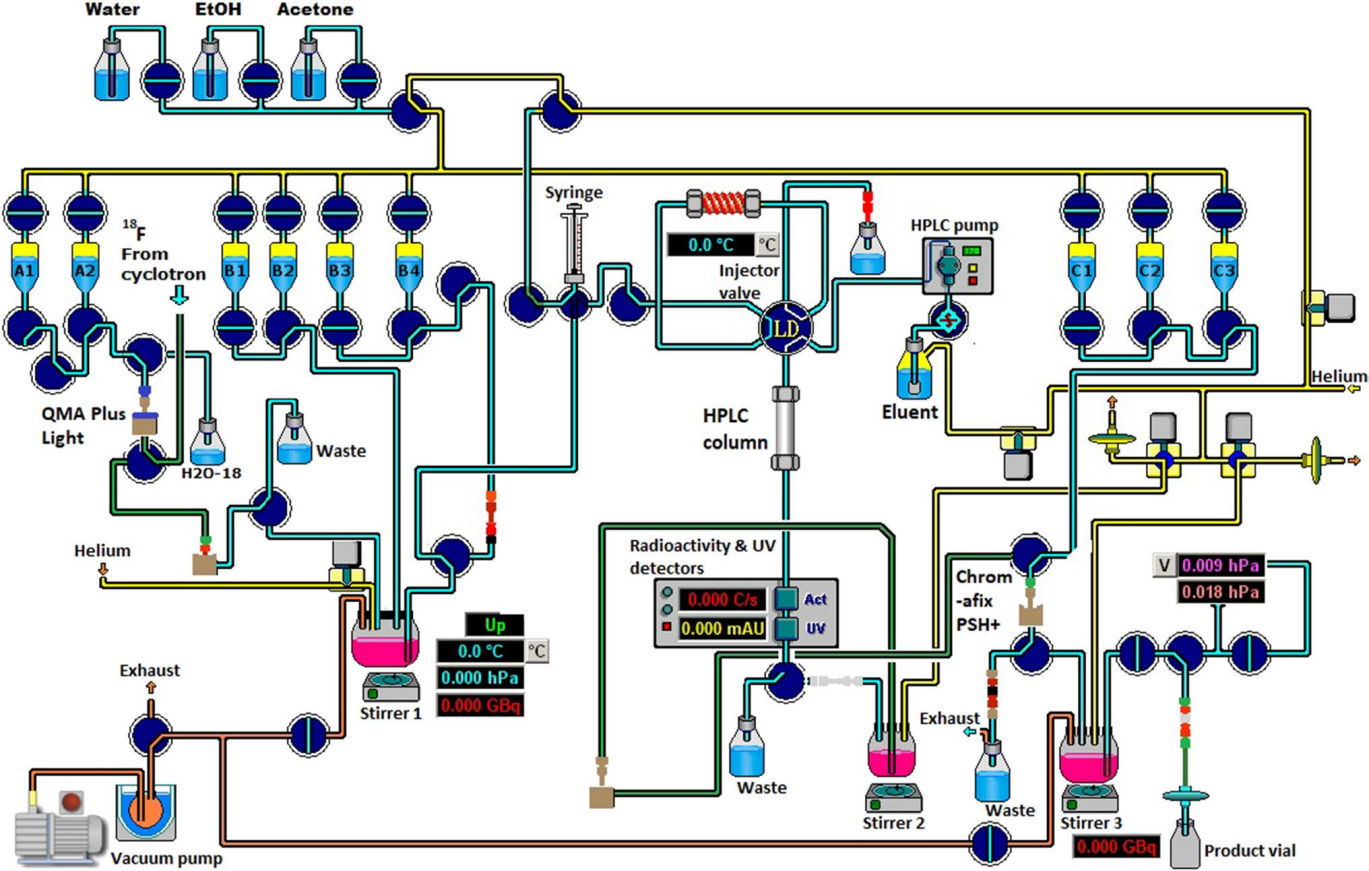
Automated system for the synthesis of [^18^F]FSPG. Reagent vials contained the following reagents: A1 = kryptofix/K_2_CO_3_ solution (1.0 mL), A2 = CH_3_CN (1.0 mL), B1 = FSPG precursor (3.0 mg) in CH_3_CN (1.0 mL), B2 = 1.0 M H_2_SO_4_ (1.0 mL), B3 = 4.0-M NaOH (0.75 mL), B4 = PBS (3.0 mL), C1 = H_2_O (6.0 mL), C2 = H_2_O (6.0 mL), C3 = PBS (3.0 mL).

The fully automated synthesis of [^18^F]FSPG was carried out on a customised RNPlusSynthra module (Fig. 2) as follows. Cyclotron produced, no-carrier-added [^18^F]fluoride was passed through a QMA SepPakPlus Light cartridge. The trapped [^18^F]fluoride was eluted from the cartridge into a reaction vessel using a 1.0-mL aliquot from a stock solution containing Kryptofix 2.2.2 (120 mg, 0.319 mmol)/K_2_CO_3_(22 mg, 0.159 mmol)/CH_3_CN (1 mL)/H_2_O (14 mL). The resultant mixture was azeotropically dried under a stream of nitrogen and vacuum at 110 °C for 8 min. A further 1 mL of CH_3_CN was added, and the mixture was dried again under a stream of nitrogen and vacuum at 110 °C for an additional 3 min. An FSPG precursor (3.0 mg) in anhydrous CH_3_CN (1.0 mL) was added to the dried [^18^F]fluoride residue, and the mixture was heated at 100 °C for 15 min. The resultant mixture was cooled to 40 °C; then, 1.0-M H_2_SO_4_ (1.0 ml) was added, and the reaction mixture was heated at 100 °C for 5 min under open vessel conditions. The resultant mixture was cooled to 40 °C; then, 4.0-M NaOH (0.75 mL) was added, and the reaction mixture was heated at 100 °C for a further 5 min under open-vessel conditions. After cooling to 40 °C, PBS (3.0 mL) was added, and the resultant mixture was injected onto an HPLC column (PhenomenexSynergi Hydro R, 4 micron, length = 150 mm, diameter = 10 mm) and eluted with 1.67% ethanol at a flow rate of 2.0 mL min^−1^. The eluent passing through the HPLC column was monitored continuously for radioactivity and absorbance at 218 nm. The fraction containing [^18^F]FSPG (retention time, *Rt* = 6.5–7.8 min) was collected and stirred in a round bottom flask containing 0.1-M H_2_SO_4_ (30 mL). After stirring, the resultant solution was passed through a Chromafix PS-H^+^ cartridge [conditioned before use by passing ethanol (10 mL) then deionised water (10 mL) through the cartridge]. The cartridge was washed with deionised water (12 mL), and the radioactive product was eluted with PBS (3.0 mL). Finally, the [^18^F]FSPG formulation was dispensed via a 0.2 micron filter into a sterile vial for preclinical application.

### Quality Control Analysis of [^18^F]FSPG

#### Chemical and Radiochemical Purity (Direct Analysis)

[^18^F]FSPG formulated in PBS (10-µL aliquot) was analysed by HPLC using a Thermo Fisher Hypercarb C_18_ column (150 × 4.6 mm) eluted at 0.9 mL min^−1^ with a mixture of 0.1% trifluoroacetic acid and acetonitrile (94:6). The eluate was monitored continuously for radioactivity and absorbance at 218 nm. The retention time for FSPG was 4.6 min. The formulated product gave one radioactive peak with the same retention time (4.6 min) as authentic FSPG. The radiochemical purity was 95–97%. For confirmation, [^18^F]FSPG formulated in PBS (50 µL) was mixed with a 50-µL sample of a 100-µM reference solution of authentic FSPG. A 10-µL aliquot of the resultant mixture was then analysed by HPLC as described above.

#### Molar Activity Determination (Analysis Using Pre-Column Derivatisation)

[^18^F]FSPG formulated in PBS (a 10-μL aliquot) was mixed with OPA reagent solution (10 μL) and stirred for 1 min at room temperature. The resultant sample was analysed by HPLC using a Waters Spherisorb C_18_, 3-µm column (250 × 4.6 mm) eluted at 1.0 mL min^−1^ with a mixture of 10-mM NaH_2_PO_4_ (pH adjusted to 7.0 using 5.0-M NaOH) and methanol (60:40). The eluate was monitored continuously for radioactivity and absorbance at 338 nm. A sample of authentic FSPG (100 µM, 50 µL) when incubated at room temperature for 1 min with 50 µL of OPA reagent gave a single peak with a retention time of 4.6 min. The derivatised^18^F-fluorinated product gave one radioactive peak and one stable peak both with the same retention time (4.6 min) as the OPA derivative of authentic FSPG. Typically, the radiochemical purity was 95–97%, and the average molar activity was 66 GBq µmol^−1^ (range, 44–73 GBq µmol^−1^) at the end of synthesis (EOS).

## Results and Discussion

This work was undertaken to support a preclinical study of the PET imaging characteristics of the transport system x_c_^−^ using [^18^F]FSPG in a mouse tumour model. The study requires that samples of [^18^F]FSPG be prepared on demand and transported off-site to an animal PET scanning facility prior to use. It was, therefore, necessary to have a reliable radiosynthesis method that was able to produce [^18^F]FSPG in high chemical and radiochemical stability at room temperature. Furthermore, the method had to yield [^18^F]FSPG in high radioactivity volumetric concentration and molar activity to provide the small volume doses needed for preclinical PET.

**Fig. 3.**
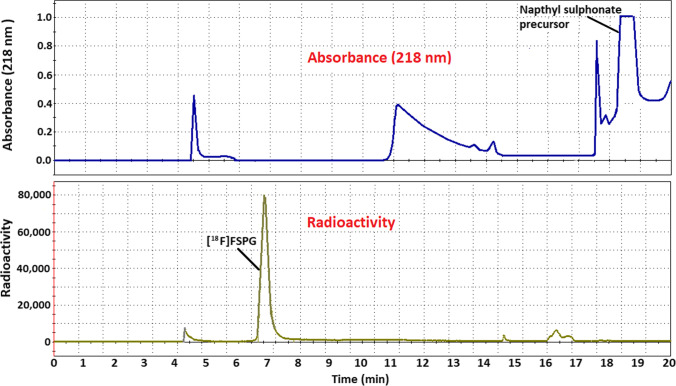
A typical HPLC chromatogram of [^18^F]FSPG purification.

**Fig. 4.**
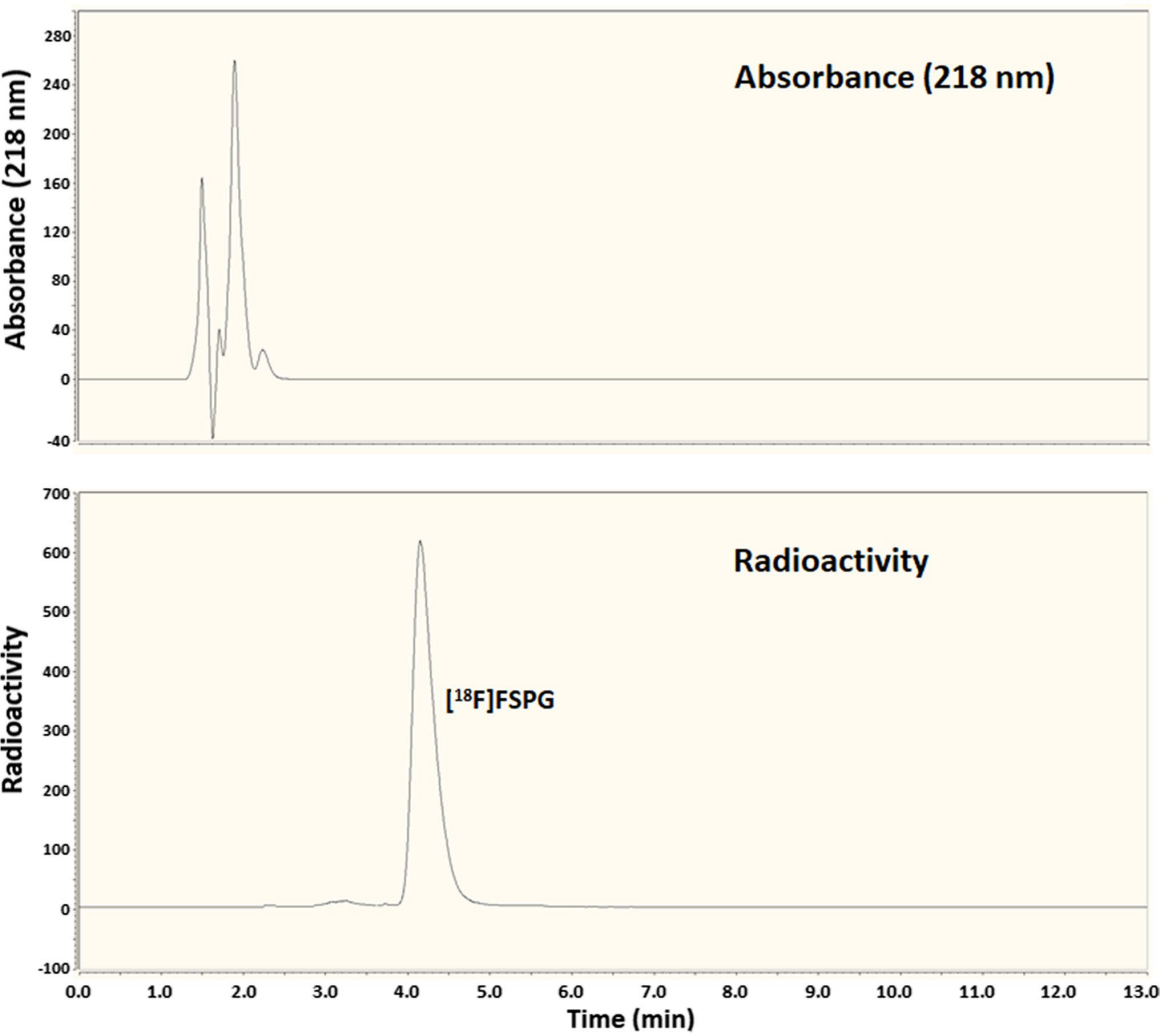
Quality control direct analysis of [.^18^F]FSPG (UV absorbance peaks at 1.0–3.0 min are attributable to PBS, pH 7.4).

The substitution reaction of nucleophilic [^18^F]fluoride with a naphthylsulfonyloxy-propyl-L-glutamate derivative, containing *tert*-butyl and trityl-protecting groups at the carboxylic *O*-hydroxy and *N*-amino groups, respectively, was used to synthesise [^18^F]FSPG labelled in the fluoropropylmoiety with fluorine-18 (Fig. 1b). The radiolabelling step was performed in acetonitrile under sealed, thermal conditions. The resultant radiolabelled product was then heated under open-vessel conditions for cleavage of *tert*-butyl and trityl-protecting groups. While still under open-vessel conditions, the reaction mixture was heated at 100 °C with concentrated sodium hydroxide. This was to degrade or remove any potential volatile by-products of the radiolabelling and deprotection steps, such as *tert*-butyl alcohol (*tert*-butanol: boiling point = 83 °C) [27], which might hinder purification of the product. For purification, semi-preparative HPLC was used to isolate [^18^F]FSPG, while the lipophilic3-(2-naphthylsulfonyl)oxy)propyl precursor was strongly retained on the HPLC column and was later eluted using 50% ethanol (Fig. 3). The [^18^F]FSPG was collected in a dilute acid solution then trapped by solid phase extraction on a cation exchange cartridge, containing a sorbent with strong anionic functional groups [28]. This ion exchange trapping process requires that [^18^F]FSPG behaves in aqueous solution as a zwitterion in the same manner as its glutamic acid parent compound [29]. Thus, at low pH, under acidic and aqueous conditions, it exists in a predominantly protonated form with a net charge of + 1 due to a positive charge at the amino group. This allows it to be trapped on the cartridge due to ionic interaction with anionic groups on the cartridge sorbent. On elution of the cartridge with PBS (pH = 7.4), the [^18^F]FSPG is converted to a deprotonated form with a net charge of − 1 due to deprotonation at both carboxylic acid groups, while the amino group retains a positive charge. In this deprotonated form, it is released from the cartridge into the PBS solution (pH = 7.4) then filtered for use in clinical and preclinical PET imaging studies. Using this method, [^18^F]FSPG was produced typically in 39–43% radiochemical yield (decay corrected) from [^18^F]fluoride. After dispensing, the average radioactivity yield of [^18^F]FSPG as formulated for preclinical use was 4.2 GBq (range, 3.4–4.8 GBq), starting from 16 GBq of [^18^F]fluoride at the end of bombardment (EOB) (*n* = 10). The average molar activity was 66 GBq µmol^−1^ (range, 44–73 GBq µmol^−1^) at EOS, and the radioactivity volumetric concentration was 343–400 MBq mL^−1^ at EOS. No chemical impurities were observed in the final product at 218-nm absorbance (Fig. 4).

Values of [^18^F]FSPG molar activity at the end of synthesis for previously reported methods (Table 1) are typically between 4 and 48 GBq µmol^−1^ [16, 17, 24–26]. These methods rely on solid phase extraction (SPE) using multiple cation exchange cartridges in series for product purification and isolation. However, SPE in this way can be problematic in terms of poor recovery, reproducibility issues, and sample extracts being insufficiently clean. There is a potential for structurally similar side products to form and co-elute as part of the final formulated product. The authors of these reported methods discuss the challenges of SPE in relation to [^18^F]FSPG purification [22, 25]. Product purification is challenging because SPE does not involve real-time analysis of the recovered eluate. In the case of the synthesis of [^18^F]FSPG, which uses harsh acidic and basic reagents, the HPLC purification method employed here offers a more accurate and reproducible way to recover products in high purity and high molar activity. This is important from a PET imaging perspective since [^18^F]FSPG is used as a transporter radioligand for the transport system x_c_^−^ [15, 16]. Glutamine and cystine are the endogenous AA targets for this transport system and so [^18^F]FSPG molar activity must be sufficiently high to avoid saturation of the transporter. Tumour cell uptake studies using L-[5-^11^C]glutamine with molar activity of 1.85 GBq µmol^−1^ at EOS have a maximum uptake of 22% in SF188 tumour cells and in dynamic small-animal PET studies using rats, bearing 9L tumor xenografts; it shows barely measurable tumour uptake and retention [30, 31]. By comparison, in the same xenograft model, [^18^F](2S,4S)-4-(3-Fluoropropyl)glutamine shows good tumour uptake compared to the muscle (background) regions [8]. PET studies using [^18^F]FSPG at the molar activity reported here could potentially allow improved tumour uptake characteristics.

**Fig. 5.**
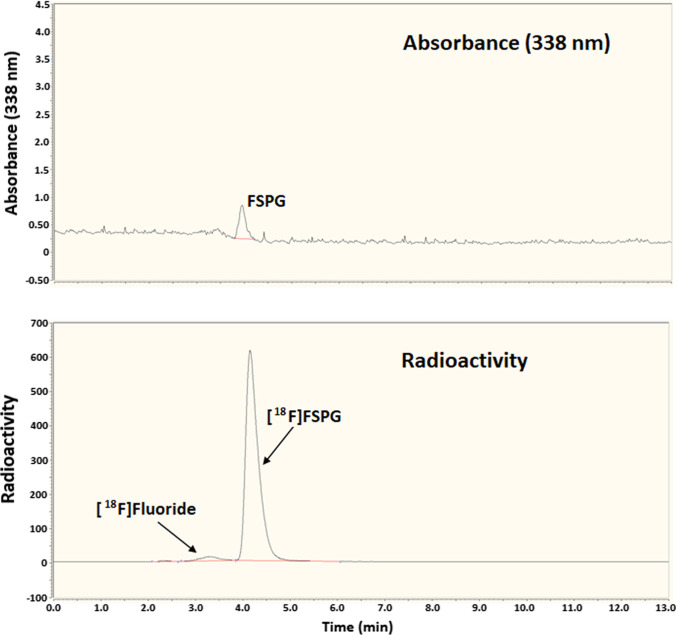
Quality control indirect analysis of [^18^F]FSPG using pre-column OPA derivatisation.

Amino acids are typically difficult to detect by UV absorbance due to a lack of a suitable UV chromophore. Pre-column derivatisation using OPA Reagent is, therefore, used as a method for indirect HPLC analysis, whereby the amino acid analyte is converted to a UV detectable isoindoline derivative. The method is used for amino acid analysis in biological, industrial, and environmental applications [32–34] where a high detection sensitivity is necessary. However, the reagent is expensive, requires careful handling, has a limited shelf life, and, crucially, it may not react with product sample impurities to produce compounds identifiable by UV detection. Despite these drawbacks, the reagent is highly sensitive and reacts with trace amounts of a wide variety of compounds containing primary amino substituents. Using this indirect method, the radiochemical purity (RCP) and molar activity (MA) of the formulated radioactive product were measured at 95–97% and 44–73 GBq µmol^−1^, respectively, with [^18^F]fluoride as the only radioactive impurity (Fig. 5). Radiostability over time was tested using an^18^Fproduct dose of 4.6 GBq with a radioactivity volumetric concentration of 369 MBq mL^−1^ at EOS. Radioanalyses of the final product at EOS and at 1.5 and 3.0 h after EOS were performed, and, at all 3 time points, the RCP was measured at 95% with [^18^F]fluoride as the only radioactive impurity. In between radioanalyses, the product was stored under sealed conditions at room temperature. For confirmation, the radiostability analysis was repeated using an alternative method, which allowed direct analysis of [^18^F]FSPG. The method used a Thermo Fisher Hypercarb C_18_ column, which has a sufficiently high carbon loading to retain hydrophilic FSPG and allow it to be eluted with a reasonable retention time (Fig. 4). Using this method, the identity of the final product was first confirmed by co-elution with an authentic FSPG reference prior to radiostability testing. The results of the radiostability analysis were the same as those of the indirect method, confirming that the product is suitable for use up to 3-h post-radiosynthesis. The direct analysis method is simpler but has a lower limit of detection for FSPG of *ca.* 50 µM and, therefore, is not sufficiently sensitive for FSPG analysis at the no-carrier-added level. This simplified procedure could be used for routine radiochemical purity and chemical purity measurements as well as radiostability testing, whereas the indirect method involving analyte derivatisation is useful for determining molar activity.

## Conclusions

FSPG was labelled in the fluoropropylmoiety at the 4-position of the glutamic acid backbone with ^18^F via the substitution reaction between nucleophilic [^18^F]fluoride and di-*tert*-butyl-(2S,4S)-2-(3-((naphthalen-2-ylsulfonyl)oxy)propyl)-4-(tritylamino)pentanedioate. Radiolabelling was achieved by a method utilising semi-preparative reverse-phase HPLC purification and solid-phase extraction. This is in contrast to previously reported methods, employing cartridge purification, which do not allow real-time analysis of the purification process and are, therefore, prone to issues with reproducibility and product separation efficiency. The method developed here delivers a product with improved molar activity at the end of synthesis compared to previously reported methods, which could potentially have better PET imaging characteristics with respect to imaging the amino acid transport system x_c_^−^. To simplify the product analysis, we have employed a direct method of radio-HPLC analysis, which avoids the need for pre-column derivatisation of the radiolabelled product using OPA Reagent, an expensive derivatising agent with limited chemical reactivity and a short shelf life. Stability tests using both direct and indirect methods of radioanalysis show that the formulated product is of high radiochemical purity (RCP > 95%), undergoes no observable radiolysis, has an acceptable range of molar activity (MA = 44–73 GBq µmol^−1^ EOS) and is suitable to inject into mice up to 3 h after production.
